# Successful management of emphysematous prostatic abscess and concurrent liver abscess: A rare case report

**DOI:** 10.1016/j.eucr.2023.102571

**Published:** 2023-09-21

**Authors:** Abdoulhafid Elmogassabi, Tawiz Gul, Bela Tallai, Maged Alrayashi, Mohamed Abdelkareem, Mohammed Ibrahim, Abu Baker, Mohammed Ebrahim, Hossameldin Alnawasra, Salvan Alhabash, Morshed Salah

**Affiliations:** a Urology Section, Surgery Department, Hazm Mebaireek General Hospital, Hamad Medical Corporation, Qatar; b College of Medicine, Qatar University, Doha, Qatar

**Keywords:** Emphysematous prostatic abscess, Concurrent liver abscess, Klebsiella pneumoniae

## Abstract

Emphysematous prostatic abscess (EPA) is a rare condition characterized by gas and abscess accumulation in the prostate. In this case report we report a successfully treated EPA with liver abscess due to Klebsiella pneumoniae in a 49-year-old man. He was admitted with abdominal pain and fever. Physical examination revealed tender, palpable resonance urinary bladder, and prostatic tenderness on rectal digital examination. High inflammatory markers were found. Abdominal computer tomography (CT) confirmed EPA. The patient was treated with broad-spectrum antibiotics, strict blood glucose control, suprapubic catheterization, and transurethral deroofing of the prostatic abscess. After three weeks patient discharged in good condition.

## 1 Introduction

Emphysematous prostatic abscess is a rare inflammatory condition characterized by gas collection and purulent exudate within the prostate. Because of its non-specific presentation and symptoms, like dysuria, frequency, urgency, fever, acute urinary retention, and perineal pain, as well as having a high mortality rate, the condition requires special attention from physicians. The mortality rate varies between 1% and 16%.^1^ The most common microorganism causing EPA with septic metastatic lesions is Klebsiella pneumoniae.^2^

## 2 Case presentation

A 49-year-old male presented to our emergency department having generalized abdominal pain, nausea, vomiting, and fever for four days, along with dysuria, urgency, and a weak urine stream. He was not known as diabetic, however, his hemoglobin A1c (HbA1c) was >12.5%.

On physical examination, the patient looked sick and lethargic, however, he was afebrile, conscious, and oriented. The abdomen was soft and lax. The urinary bladder was palpable up to the umbilicus and resonance was heard on percussion. A digital rectal examination demonstrated an enlarged, tender prostate. His blood pressure was 110/80 mmHg; pulse rate was 100/min, body temperature was 37 °C, with a respiratory rate of 18/min. Laboratory investigations revealed high white blood cells 29x10³/μl, along with urea 12.4 mmol/l, creatinine 113 μmol/l, CRP 234 mg/l, HB A1c > 12%, albumin 23 gm/L, alkaline phosphatase 262 U/L, alanine aminotransferase (ALT) 32 U/L and aspartate aminotransferase (AST)18 U/L. Midstream urine culture was positive for Klebsiella pneumoniae.

**Fig. 1:**
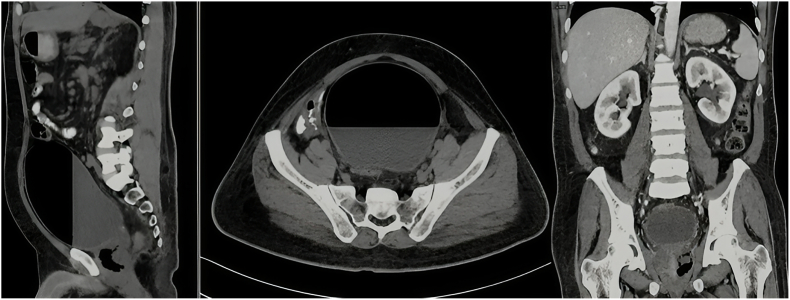
CT abdomen and pelvis with contrast revealed a multiloculated prostatic abscess measuring about 5 x 6 × 5.6 cm with intraprostatic gas collections and an air-fluid level in the urinary bladder.

Intravenous contrast-enhanced abdominal and pelvic computed tomography (CT) revealed a multiloculated prostatic abscess measured at about 5 x 6 × 5.6 cm (87 ml) with intraprostatic gas collections in addition to an air-fluid level in the urinary bladder (Fig. 1) together with evidence of right liver lobe abscess.

Based on our local antibiotic protocol, broad-spectrum empiric antibiotic treatment was initiated, with the administration of intravenous piperacillin/tazobactam (4.5 g) every 8 hours.

Urgent transurethral deroofing of the prostatic abscess, and simultaneous suprapubic catheter (SPC) insertion were performed. Twenty French 3-way Foley catheter was left for postoperative temporary irrigation.

On day 1, post-surgery, the patient's general condition improved, and no fever was recorded. His laboratory parameters also improved. Parenteral metronidazole and strict control of high blood glucose were initiated. On day 2, the foley catheter was removed. His ongoing antibiotics were approved by the infectious disease team (ID). The possibility of intervention for the liver abscess provided no improvement with antimicrobial therapy, was also mentioned from their side. On day 3, the histopathology report confirmed the prostatic abscess. On day 7, metronidazole was stopped. On day 8, the patient's condition deteriorated, he became febrile, and his inflammatory markers started to rise again. Thereafter, the ID team upgraded his antimicrobial treatment to meropenem 1 gm, to be administered every 8 hours.

**Fig. 2:**
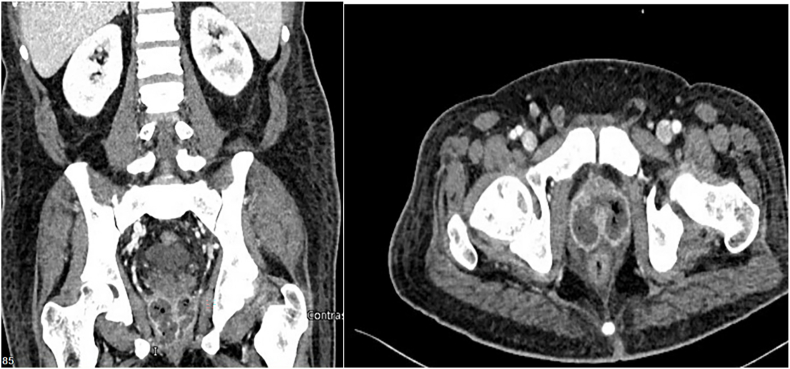
CT abdomen and pelvis with contrast ten days after the 1st resection showed a slight interval reduction in the collection size within the prostatic gland.

On day 10, the patient's general condition did not improve with having spikes of fever. (39° C). The white blood cells reached 19.3x10³/μl and CRP also raised to 132 mg/l. CT abdomen and pelvis with intravenous contrast was repeated (Fig. 2), showing a significant residual prostatic collection with gases measured as 5.4 × 5.3 × 4.5 cm. Therefore, second transurethral prostate abscess deroofing with aggressive re-resection was indicated and carried out, almost reaching the prostatic capsule, especially at the right lobe. More purulent material was drained out, along with a lot of necrotic tissue around the abscess cavity.

**Fig. 3:**
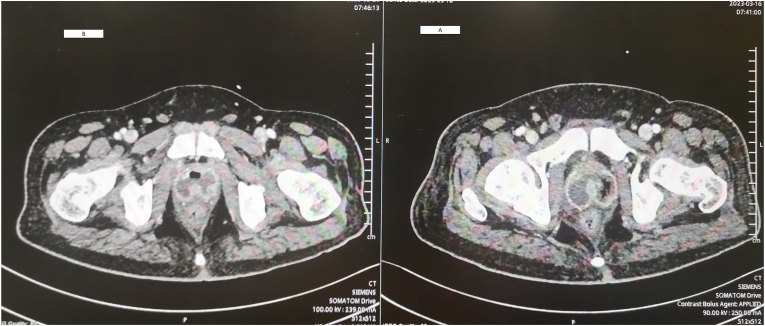
Repeated CT abdomen and pelvis with contrast showed the difference between a. After the first and b. After the second resection.

After the second transurethral resection of the prostate, computed tomography showed remarkable regression in prostatic abscess compared to the previous one (Fig. 3). The inflammatory parameters improved gradually, although the patient developed fever spikes over six days in the postoperative period. On day 18, no more fever was recorded, inflammatory markers declined to normal levels, and the patient became completely asymptomatic.

On day 24, SPC was removed, and he was discharged in good condition on trimethoprim/sulfamethoxazole 960 mg tablet q 12 hourly for three weeks.

## 3 Discussion

Prostatic abscess is a rare complication of acute bacterial prostatitis, reported in 0.5%–2.5% of patients presenting with inflammatory prostatitis.^3^ An emphysematous prostatic abscess is a sporadic form of a prostatic abscess, characterized by a localized collection of gas and purulent exudate inside the prostate gland.^4^ It is a severe gas-forming infection of the urinary tract. The mortality rate of EPA is approximately 25%, higher than that of emphysematous cystitis (7%) or emphysematous pyelonephritis (21%).^5^ The presenting symptoms and signs of EPA are variable, including dysuria, fever, urinary frequency and urgency, urinary retention, and perineal pain. The early diagnosis is difficult due to nonspecific symptoms; for that reason, many patients are diagnosed late.^6^ Imaging techniques like contrast-enhanced pelvic CT and transrectal ultrasonography (TRUS) are the most valuable diagnostic tools for the early detection of EPA.

Diabetic patients are more prone to complicated urinary tract infections, like EPA. The other risk factors include infra-vesical obstruction, neurogenic bladder, urethral instrumentation, liver cirrhosis, and post-renal transplant status.^5^ Therefore, we should raise high suspicion for such severe urinary tract infections when there is no good response to medical treatment, especially in those having risk factors.

Patients with a known history of diabetes mellitus have a high incidence of bacteriuria. Diabetes mellitus with urinary tract infections and obstruction can be predisposing factors leading to emphysematous infections of the genitourinary tract. Based on that, we can also assume that undiagnosed DM along with uncontrolled blood sugar are also significant risk factors. So far, only a few emphysematous prostatic abscesses have been reported in the literature, and no single case has been associated with concomitant liver abscesses.

Once EPA is suspected, contrast-enhanced CT provides the most accurate diagnosis. Early detection, prompt drainage, and sufficient antibiotic treatment are crucial to avoid this severe infection's potential morbidity and mortality.

K. pneumoniae has been reported to be the most common pathogen responsible for metastatic infection.^7^ However, other organisms, like Escherichia coli, Proteus mirabilis, Citrobacter species, Pseudomonas aeruginosa, Bacteroides fragilis, and yeasts have also been reported.^8^ In some cases, multiple organs are infected, and the presentation depends upon which organ is more affected. For example, some investigators have reported that a minority of patients with Klebsiella liver abscess, and mainly from Taiwan, develop a metastatic infection as manifested by endophthalmitis, uveitis, pneumonia, lung abscess, pulmonary emboli, pleural empyema, peritonitis, subcutaneous abscess, deep neck infection involving the mediastinum, splenic abscess, brain abscess, purulent meningitis, epidural abscess, renal abscess, prostate abscess, osteomyelitis, pyogenic arthritis, and psoas abscess.^7^, ^8^, ^9^ This disease called invasive liver abscess syndrome caused a hypervirulent K. pneumoniae subtype.^9^, ^10^, ^11^ Recent studies have shown that the mucoviscosity-associated gene A (magA) and regulator of mucoid phenotype A (rmpA) are associated with this syndrome.^7^ In our patient, the primary site of the abscess cannot be ascertained. Was it a liver abscess with metastatic infection to the prostate or vice versa? In any way, the approach and the proper management are the same.

The proper management of the EPA needs multidisciplinary teamwork. As we did with our patient, the urologist, internal medicine, infectious team, and radiologist were involved in the treatment plan. However, the cornerstone of treatment was adequate abscess drainage with early antibiotic therapy and strict blood glucose control. In the case of EPA, abscess drainage can be performed by TRUS-guided aspiration, transurethral incision or deroofing, or *trans*-perineal approach. TRUS-guided aspiration drainage is considered the standard treatment before progressing to other therapies.^12^ Transurethral incision or unroofing can provide complete drainage but also increase the risk of sepsis. Hydraulic pressure during the transurethral procedure may push pathogens into the systemic bloodstream, leading to sepsis or septic shock. Therefore, it should be done in selected patients who are hemodynamically stable and fit for the anaesthesia, and it is best when it is quick and effective. In the case we presented, TRUS-guided aspiration was inadequate for abscess drainage because the lesion was multilocular. We also assumed that the air, the thick purulent exudate, and the necrotic tissue of EPA made it challenging to drain by aspiration, and many patients might have a much higher chance to recur (one resection was not enough for our patient due to the multilocularity).

## 4 Conclusion

Emphysematous prostatic abscess combined with a liver abscess is a rare but potentially life-threatening complication of acute bacterial prostatitis. Early recognition and appropriate management are critical to reduce morbidity and mortality. Clinicians should consider this diagnosis in patients with potential risk factors like DM associated with urinary tract infections, not responding well enough to conventional management. With our case presentation, our intention was to call the attention of that rare entity in patients having non-specific symptoms and septic parameters.
